# Population-based survival estimates for childhood cancer in Australia during the period 1997–2006

**DOI:** 10.1038/sj.bjc.6605985

**Published:** 2010-11-09

**Authors:** P D Baade, D R Youlden, P C Valery, T Hassall, L Ward, A C Green, J F Aitken

**Affiliations:** 1 Viertel Centre for Research in Cancer Control, Cancer Council Queensland, 553 Gregory Terrace, GPO Box 201 Spring Hill, Fortitude Valley QLD 4006 Australia; 2 School of Public Health, Queensland University of Technology, Victoria Park Road, Kelvin Grove QLD 4059 Australia; 3 Queensland Institute of Medical Research, 300 Herston Road, Herston QLD 4006 Australia; 4 The Australian Centre for International and Tropical Health, University of Queensland, Herston Road, Herston QLD 4006 Australia; 5 Royal Children's Hospital, Herston Road, Herston QLD 4006 Australia; 6 University of Manchester, Oxford Road, Manchester M13 9PT, UK; 7 School of Population Health, University of Queensland, Herston Road, Herston QLD 4006, Australia

**Keywords:** cancer survival, paediatric, childhood, population-based, leukaemia, lymphoma

## Abstract

**Background:**

This study provides the latest available relative survival data for Australian childhood cancer patients.

**Methods:**

Data from the population-based Australian Paediatric Cancer Registry were used to describe relative survival outcomes using the period method for 11 903 children diagnosed with cancer between 1983 and 2006 and prevalent at any time between 1997 and 2006.

**Results:**

The overall relative survival was 90.4% after 1 year, 79.5% after 5 years and 74.7% after 20 years. Where information onstage at diagnosis was available (lymphomas, neuroblastoma, renal tumours and rhabdomyosarcomas), survival was significantly poorer for more-advanced stage. Survival was lower among infants compared with other children for those diagnosed with leukaemia, tumours of the central nervous system and renal tumours but higher for neuroblastoma. Recent improvements in overall childhood cancer survival over time are mainly because of improvements among leukaemia patients.

**Conclusion:**

The high and improving survival prognosis for children diagnosed with cancer in Australia is consistent with various international estimates. However, a 5-year survival estimate of 79% still means that many children who are diagnosed with cancer will die within 5 years, whereas others have long-term health morbidities and complications associated with their treatments. It is hoped that continued developments in treatment protocols will result in further improvements in survival.

More than 600 children between 0–14 years of age are diagnosed with cancer each year in Australia corresponding to an age-standardised incidence rate (2000 World Standard) of 157 cases/million children per year (Baade *et al*, 2010). This incidence rate is among the highest reported internationally (Steliarova-Foucher *et al*, 2005a; Stack *et al*, 2007; Li *et al*, 2008; Linabery and Ross, 2008; Ocheni *et al*, 2008; Spix *et al*, 2008; Swaminathan *et al*, 2008), consistent with the strong positive association between country-specific childhood-cancer incidence rates and affluence (Howard *et al*, 2008). Although the Australian childhood cancer incidence rate represents less than 1% of all invasive cancers diagnosed in this country, it is still the most common cause of disease-related death for children between 1–14 years of age in Australia (Australian Institute of Health and Welfare, 2009). Many survivors face significant long-term adverse health effects because of the cancer itself or as a result of treatment (Aziz *et al*, 2006; Goldsby *et al*, 2006; Kurt *et al*, 2008; Landier and Bhatia, 2008; Oeffinger *et al*, 2008). The diagnosis of cancer in a child also has a considerable and ongoing impact, both psychological and financial, on the families concerned (Cohn *et al*, 2003; Hardy *et al*, 2008).

Advances in therapy for childhood cancer, including the introduction of multiagent chemotherapy and multimodal therapy, combined with greater understanding of the molecular basis of childhood cancers, have led to widespread improvements in survival for childhood cancer in developed nations. (Zuccolo *et al*, 2006; Ellison *et al*, 2007; Anonymous, 2009; Perme and Jereb, 2009).

Ongoing population-based survival studies are essential for providing robust indicators to monitor the availability of effective treatments and healthcare provision for cancer patients, and to compare the cancer burden between countries (Gatta *et al*, 2002; Desandes *et al*, 2008). Although some survival estimates for childhood cancers have been published recently for Australia (AIHW and AACR, 2008), the use of the standard site-based ICD-0-3 classification has limited comparisons with international estimates that use the current morphology-based standard for coding childhood cancer, the third edition of the International Classification of Childhood Cancers (ICCC-3) (Steliarova-Foucher *et al*, 2005b).

This paper reports the latest available population-based survival estimates for children diagnosed with cancer in Australia categorised by the ICCC-3 diagnostic groupings, enabling for the first time comparability with population-based estimates from other countries.

## Materials and methods

### Australian paediatric cancer registry

The Australian Paediatric Cancer Registry (APCR) is one of the few population-based national registries of childhood cancer in the world. Established in 1977, it obtained full coverage of all Australian states and Territories from 1983, and currently includes cases upto 31st December 2006. Notification of invasive cancer is a statutory requirement for all public and private hospitals and pathology services in Australia, and so the survival data reported here are considered to represent all eligible Australian children between 0–14 years of age diagnosed with invasive cancer. Confirmation and validation of cancer records are achieved through site visits by the APCR Data Manager to the major children's hospitals around Australia, when patients’ charts are reviewed and additional information on clinical characteristics and treatment are extracted. Since 1983, 95.3% of diagnostic records in the APCR were based on histological verification (74.0% histology of primary, 0.3% histology of metastasis, 20.7% on cytology or Haematology and 0.4% on autopsy with histology) (Baade *et al*, 2010). Of the remainder, most were clinical investigations (3.9% of total). Less than 0.2% of diagnoses were based on death certificate only.

Although tumours of benign or uncertain behaviour are generally not reported for adults, the ICCC-3 includes non-malignant intracranial and intraspinal tumours in diagnostic groups III (tumours of the central nervous system) and X (germ cell tumours – see Table 1) (Steliarova-Foucher *et al*, 2005b). Therefore, throughout this paper, childhood cancers refer to all malignant neoplasms as well as intracranial and intraspinal tumours of benign or uncertain behaviour. Survival results are reported here by ICCC-3 diagnostic groups, with additional results provided for diagnostic subgroups where numbers were sufficient for meaningful interpretation.

### Mortality status

Follow-up for mortality status upto the 31st December 2006 was performed through record linkage between the APCR database and the Australian National Death Index. The record linkage, using deterministic and probabilistic algorithms, was undertaken by staff at the Australian Institute of Health and Welfare.

### Relative survival

Relative survival was used to approximate disease-specific survival because it does not rely on accurate cause of death coding (Dickman *et al*, 2004). It was calculated from the observed probability of all-cause survival among childhood cancer patients divided by the expected probability of survival within the corresponding Australian population stratified by age, sex and calendar year. Relative survival estimates were calculated using actuarial techniques based on the period methodology (Brenner *et al*, 2004). The period method has been shown through validation studies to be particularly useful in monitoring childhood cancer survival and provides more timely estimates of survival than the cohort method (Brenner *et al*, 2007; Steliarova-Foucher *et al*, 2007).

Using the period method, cancer patients were considered ‘at risk’ of mortality if they constituted a prevalent case for at least some time during the 10-year period from 1st January 1997 to 31st December 2006. The survival times of patients who were not known to have died before 31st December 2006 were censored at that date. The Ederer II method (Ederer *et al*, 1961) was used to calculate expected survival. Cases diagnosed on the basis of death certificate only (*n*=21, 0.15%) and autopsy with histology (*n*=82, 0.58%) were excluded from the survival analysis.

### Stage (specific cancers only)

Information about the spread of disease at diagnosis was collected through patients’ clinical records for the following diagnostic groups: lymphomas, neuroblastoma, renal tumours and rhabdomyosarcomas (a subgroup of soft-tissue sarcomas). The specific classification systems used for categorising stage for each of the diagnostic group/subgroup were: Hodgkin lymphoma – Ann Arbor classification system; (Carbone *et al*, 1971) Non-Hodgkin lymphomas (including Burkitt lymphoma) – Murphy classification system; (Murphy *et al*, 1989) Neuroblastoma – International Neuroblastoma Staging System; (Brodeur *et al*, 1993) Renal tumours – Third National Wilms’ Tumor Study; (D’Angio *et al*, 1989) and Rhabdomyosarcomas – Intergroup Rhabdomyosarcoma Study-I. (Maurer *et al*, 1988) Although there was stage information collected for some cases of retinoblastoma, the low proportion of these cancers with stage information (∼33%) was not sufficient to report. Due to small numbers, the staging categories for rhabdomyosarcomas were collapsed to I/II and III/IV for analysis.

Generally, Stage I tumours are localised to the part of the body where the cancer originated without any evidence of spread and were able to be surgically removed. Stage II tumours are similar, except that the tumour has been incompletely removed. Stage III tumours have greater regional involvement, preventing surgical resection and often including involvement of lymph nodes. Finally Stage IV tumours are when the cancer has spread (metastasised) to distant parts of the body, such as the lungs or bone marrow.

### Poisson models

Generalised linear models with a Poisson error structure were used to model the excess mortality (upto 5 years after diagnosis) associated with a diagnosis of childhood cancer for all cases combined and within each diagnostic group, including the effects of age group, sex, grouped year of diagnosis and, where relevant, stage at diagnosis. We were only able to apply the Poisson models to the diagnostic subgroups of lymphoid leukaemias (IA), acute myeloid leukaemias (IB) and rhabdomyosarcomas (IXA) because of instability and lack of convergence in the models for the other cancer subgroups.

## Results

### Description of cohort

A total of 11 903 children between 0–14 years of age who were diagnosed with cancer in Australia between 1983 and 2006 were ‘at risk’ between 1997 and 2006, with a median follow-up time of 8.9 years (range 0–24 years). Of these children, 5% (*n*=565) had died within 1 year, and 11% (*n*=1266) within 5 years of diagnosis. The most common cancers were leukaemias (32%, with 81% of these being lymphoid leukaemias), tumours of the central nervous system (CNS, 22%) and lymphomas (10%), which in combination represented nearly two-thirds (64%) of all cases.

### 1- and 5-year relative survival

The relative survival for all children diagnosed with cancer was 91% (95% CI=90–91%) after 1 year and 80% (79–81) after 5 years (Table 1). Among the diagnostic groups, 5-year survival was highest for retinoblastoma (98% (95–100)), other malignant epithelial neoplasms and melanomas (93% (90–96)) and lymphomas (90% (87–92)). The diagnostic groups with the poorest survival outcomes after 5 years were neuroblastoma (68% (63–72)), malignant bone tumours (69% (63–74)) and tumours of the CNS (71% (69–73)). Five-year survival for lymphoid leukaemias (85% (83–87)) was substantially higher than that for acute myeloid leukaemias (63% (58–68)). Among lymphomas, 5-year survival was better for Hodgkin lymphomas (98% (95–99)) than for either Burkitt lymphoma (90% (84–94)) or other non-Hodgkin lymphomas (82% (76–86)). Among children diagnosed with cancers of the CNS, 5-year survival for those diagnosed with astrocytomas (79% (76–82)) was higher than for intracranial and intraspinal embryonal tumours (49% (43–55)).

### Survival by stage at diagnosis

When children diagnosed with lymphomas, neuroblastoma, renal tumours and rhabdomyosarcomas were considered together (*n*=2896), there were 23% stage I, 17% stage II, 25% stage III and 19% stage IV cases, whereas 16% of these cancers had unknown stage (Table 2).

Of the cancers for which stage data were available, survival was significantly worse within each diagnostic group for children with more advanced stage at diagnosis (Tables 2 and 3). The largest difference in survival by stage occurred for children with neuroblastoma, where 5-year relative survival was 96% (86–99) for stage I disease compared with 49% (42–56) for stage IV disease. Although the association with stage was significant for renal tumours, the survival differential was limited to stage IV disease (Table 3).

### Survival by sex

After adjustment for age-group, year of diagnosis and (where relevant) stage, the only diagnostic group for which there was a significant sex differential in relative survival was leukaemias, where girls were significantly less likely (HR=0.76; (0.62–0.93), *P*=0.007) than boys to die within 5 years of diagnosis (Table 3). When leukaemias were analysed separately by subgroup, the sex differential was significant for lymphoid leukaemias (HR=0.65; (0.50–0.85), *P*=0.002), but not for myeloid leukaemias (HR=1.04; (0.72–1.49), *P*=0.840).

### Survival by age-group

For those cancers for which there was a significant age differential in survival outcomes (after adjusting for sex, year of diagnosis and, where relevant, stage), survival was generally poorer for very young cancer patients (infants diagnosed at less than 1 year), and also, to a lesser extent, for older children (5 years and over) compared with children between 1–4 years of age at diagnosis (Table 3). The significant age differential in survival for all childhood cancers combined (*P*=0.002) was largely because of the poorer survival among infants (HR=1.39; (1.2–1.7), *P*=0.001). When analysed by diagnostic group, there was a poorer prognosis among infants for all leukaemias combined (*P*<0.001). This was particularly evident for lymphoid leukaemias (*P*<0.001), whereas the age effect was not significant for myeloid leukaemias (*P*=0.123). In addition, infants had poorer survival for tumours of the CNS (*P*<0.001) and renal tumours (*P*<0.05); however, infants had significantly better survival for neuroblastoma (*P*<0.001). Older children also had poorer survival for all leukaemias combined relative to the 1–4 year age-group (*P*<0.001), which was again limited to lymphoid leukaemias (*P*<0.001), as well as hepatic tumours (*P*<0.05), malignant bone tumours (*P*<0.05) and the subgroup of rhabdomyosarcomas (*P*<0.05). In contrast, children between 10–14 years of age had improved survival for tumours of the CNS compared with children between 1–4 years of age (*P*<0.001).

### Survival by period of diagnosis

The prognosis for all childhood cancers combined improved by grouped year of diagnosis (Table 3), with overall 5-year relative survival of 77% (76–79) for children diagnosed in 1992–1998 increasing to 81% (80–82) for children diagnosed in 1999–2006 (HR=0.84; (0.74–0.94), *P*=0.003). This improvement in survival was particularly evident among children diagnosed with leukaemias (75 *vs* 84%, respectively; HR=0.59; (0.48–0.73), *P*<0.001), and this change was consistent for both lymphoid leukaemias and myeloid leukaemias. Consistent changes were observed among boys and girls, with no evidence (*P*>0.10) of interaction between sex and time period (results not shown). There was some suggestion, although not quite reaching statistical significance, of an improvement in survival for lymphomas (86% (80–91) *vs* 91% (87–93), respectively; HR=0.61; (0.36–1.04), *P*=0.07058). Similar patterns were seen for several of the other diagnostic groups, but none of the remaining hazards ratios, including that for all cancers combined excluding leukaemia, were statistically significant (*P*>0.05).

### Long-term survival

**Figure 1:**
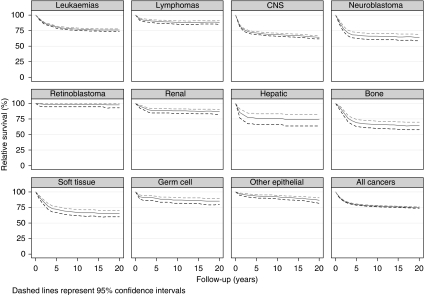
Long-term survival patterns for children diagnosed with cancer in Australia by diagnostic group, 1997–2006.

**Figure 2:**
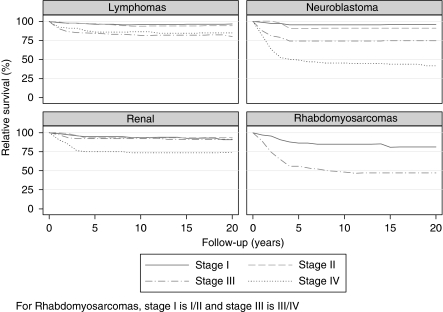
Long-term survival patterns by stage for children diagnosed with selected cancers in Australia, 1997–2006.

Long-term survival curves by diagnostic group and stage are shown in Figures 1 and 2, respectively. Overall 20-year relative survival for all children diagnosed with cancer was 75% (74–76) (Table 1). Retinoblastoma (98% (93–100)), lymphomas (88% (85–91)) and renal tumours (87% (83–90)) were the diagnostic groups with the best long-term prognoses. Of the lymphomas, Hodgkin lymphomas (95% (91–98)) and Burkitt lymphoma (91% (84–95)) had better long-term survival than other non-Hodgkin lymphomas (80% (73–85)). Cancers with the poorest long-term survival were malignant bone tumours (64% (58–70)), tumours of the CNS (64% (62–67)) and neuroblastoma (64% (59–69)).

As in the analysis of survival by stage, longer-term survival remained consistently poorer among children with more advanced cancers for each of the diagnostic groups/subgroups shown in Figure 2. However, irrespective of stage, the survival rates generally stabilised within the first few years following diagnosis.

## Discussion

This paper reports the latest survival information for Australian children diagnosed with cancer between 0–14 years of age, using a population-based paediatric cancer registry and the current international classification for childhood cancers. Analyses indicate that risk of dying within 5 years has decreased by about 16% since the early-mid 1990s, and this improvement is particularly evident for leukaemias, for which the hazard ratio was 40% lower for cases diagnosed in the late 90s or early 2000 s. Similar improvements in leukaemia survival over time have been reported in the United States (Pulte *et al*, 2008), Canada (Ellison *et al*, 2007) and France (Desandes *et al*, 2008).

Direct comparisons with published survival rates internationally need to be made with caution because of the different methodologies used, such as the period or cohort method, and the different time periods considered in the analyses. However this study has demonstrated that the 5-year survival for all childhood cancers in Australia (79%) is similar to that reported in many other developed areas of the world. Published international estimates range from 81% in 1996–2004 for the USA (Anonymous, 2009), 81% among European children in 1995–2002 (Gatta *et al*, 2009), 80% between 1998–2002 in Slovenia (Perme and Jereb, 2009), 80% in Italy between 1997–2001 (Zuccolo *et al*, 2006), 75% in France between 1990–1999 (Desandes *et al*, 2008) and 82% in Canada between 1993–2003 (Ellison *et al*, 2007). The lack of consistency in methodology when generating and reporting international estimates of childhood-cancer survival could lend support for a similar collaborative comparative study equivalent to the CONCORD study for adults (Coleman *et al*, 2008).

Children who were diagnosed when less than 1 year of age had a poorer prognosis for leukaemias (particularly lymphoid leukaemias) and tumours of the CNS, but had better prognosis for neuroblastoma. These age effects were similar to those reported in United States (Linabery and Ross, 2008), France (Desandes *et al*, 2008) and Canada (Ellison *et al*, 2007). It can be more difficult to treat younger children because of a combination of differences in the biological characteristics of their cancers along with their ability to cope with the therapies that are usually applied. For example, infants with leukaemia have been shown to be more resistant to certain types of chemotherapy (Pieters *et al*, 2007) and they can also be at higher risk of drug-induced toxicity due to slower clearance rates of some chemotherapeutic agents (Koren and Schechter, 2007). The use of traditional radiation therapy, which is widely used in the treatment of older children with a tumour of the central nervous system, is often deferred or avoided in infants because of the possibility of significant adverse late effects (Lafay-Cousin and Strother, 2009). The inverse relationship between age at diagnosis and survival for children with neuroblastoma has been reported previously, with patients less than 1 years of age at diagnosis generally having smaller and less aggressive tumours (van Noesel and Versteeg, 2004; Gutierrez *et al*, 2007; Haupt *et al*, 2010).

Overall survival was higher for children diagnosed in more recent years, however, when analysed by diagnostic group, this result was only statistically significant for leukaemias, with some suggestion of an improvement for lymphomas. Improvements in leukaemia survival over time have been reported internationally (Brenner *et al*, 2007; Gatta *et al*, 2009) and have been suggested to be most likely because of major progress in treatment regimens (Brenner *et al*, 2007) and as a direct result of collaborative clinical trials (Bond and Pritchard, 2006; O’Leary *et al*, 2008). In Australia, all the main paediatric oncology centres are publicly funded, which means there is no restriction to treatment options according to whether a child's family have private health insurance. In addition, all Australian centres have strong international collaborative connections with both USA (Children's Oncology Group) (O’Leary *et al*, 2008) and Europe (SIOP – International Society of Paediatric Oncology) (Pritchard-Jones, 2008).

As is the case for adult cancers, stage is a key prognostic factor for childhood cancer. Children with cancers that were more advanced at diagnosis generally experienced significantly poorer survival, similar to findings in the French study (Desandes *et al*, 2008). The greatest variations in survival by stage in our data were observed for neuroblastoma.

We also found that survival rates tended to stabilise within a few years after diagnosis for most of the diagnostic groups regardless of stage at diagnosis. This needs to be interpreted in the context of the quality of life of longer-term survivors of childhood cancer, which is often affected by complications such as subsequent cancers, organ dysfunction (including cardiopulmonary, renal and gastrointestinal), impaired growth and development, decreased fertility and neurocognitive deficits, some of which will not become apparent until many years later (Goldsby *et al*, 2006; Landier and Bhatia, 2008). Long-term, multidisciplinary monitoring of survivors is important in order to minimise where possible the impact of these potential, adverse effects (Hewitt *et al*, 2003; American Academy of Pediatrics Section on Hematology/Oncology Children's Oncology Group, 2009).

A strength of the Australian Paediatric Cancer Registry is its complete population coverage, because of mandatory notification of all cancers in Australia. However, even with the full population coverage in Australia, the small numbers of some types of childhood cancers diagnosed over the study period meant that the corresponding confidence intervals were relatively wide. In addition, the published values for death certificate only and histological verification are indicative of the high data quality that is achieved in Australian state and territory cancer registries (Baade *et al*, 2010). The use of the period method for the analysis of cancer survival is becoming more common and, following validation in several studies, has been recommended as the method of choice to monitor population-based survival (Brenner *et al*, 2007).

These survival estimates provide quantitative data describing the prognosis for Australian children diagnosed with cancer and demonstrate consistency with survival outcomes reported internationally. They also provide a valid, high-quality baseline of survival outcomes against which changes in survival can be monitored over time. It is hoped that with further developments in treatment protocols through large multicentre studies the improvements in survival will continue.

## Figures and Tables

**Table 1:** Relative survival for Australian children diagnosed with cancer by diagnostic group, 1997–2006^a^

		**Relative survival (95% confidence intervals)**		
**Diagnostic group**	***N* b**	**1 year**	**5 year**	**20 year**
*All cancers*	11 903	90.6 (89.8–91.3)	79.6 (78.5–80.6)	74.8 (73.7–76.0)
I. Leukaemias	3833	92.8 (91.6–93.8)	80.6 (78.8–82.2)	75.9 (73.9–77.8)
IA Lymphoid leukaemias	3121	96.3 (95.3–97.1)	85.0 (83.1–86.7)	79.7 (77.5–81.7)
IB Acute myeloid leukaemias	537	79.2 (74.5–83.2)	63.4 (57.9–68.4)	62.3 (56.7–67.5)
				
II. Lymphomas	1220	93.5 (91.3–95.2)	89.8 (87.1–92.0)	88.0 (84.8–90.6)
IIA. Hodgkin lymphomas	500	98.8 (96.4–99.6)	97.7 (94.7–99.0)	95.4 (91.4–97.8)
IIB. Non-Hodgkin lymphomas (excl. Burkitt lymphoma)	486	90.2 (85.4–93.4)	81.7 (76.0–86.1)	79.5 (73.2–84.6)
IIC. Burkitt lymphoma	206	90.3 (83.5–94.4)	90.4 (83.6–94.4)	91.2 (84.3–95.3)
				
III. Tumours of the CNS^c^	2562	82.6 (80.5–84.5)	71.0 (68.5–73.3)	64.4 (61.6–67.0)
IIIA. Ependymomas and choroid plexus tumours	242	88.8 (81.8–93.2)	69.3 (60.6–76.4)	62.2 (53.2–69.9)
IIIB. Astrocytomas	1266	87.3 (84.4–89.6)	78.8 (75.5–81.8)	71.7 (67.6–75.4)
IIIC. Intracranial & intraspinal embryonal tumours	437	70.0 (64.1–75.1)	48.7 (42.6–54.5)	41.7 (35.5–47.7)
IIID. Other gliomas	267	65.2 (57.4–71.9)	55.1 (47.1–62.3)	48.9 (40.6–56.7)
				
IV. Neuroblastoma	650	87.8 (84.0–90.8)	67.8 (62.7–72.4)	64.2 (58.7–69.1)
V. Retinoblastoma	348	99.6 (95.6–100.0)	98.4 (94.5–99.7)	97.8 (92.8–99.7)
VI. Renal tumours	683	95.5 (92.7–97.3)	88.6 (84.6–91.6)	86.9 (82.5–90.4)
VII. Hepatic tumours	153	85.5 (76.7–91.2)	76.0 (66.0–83.4)	74.4 (63.8–82.4)
VIII. Malignant bone tumours	494	93.6 (89.6–96.0)	68.9 (62.8–74.2)	64.1 (57.7–69.8)
IX. Soft tissue sarcomas	693	90.6 (87.0–93.3)	72.1 (67.0–76.6)	65.2 (59.8–70.1)
IXA. Rhabdomyosarcomas	343	92.8 (87.6–95.9)	71.7 (63.9–78.2)	65.3 (57.2–72.2)
X. Germ cell tumors^c^	467	92.2 (88.0–95.0)	89.4 (84.7–92.7)	85.0 (79.2–89.3)
XI. Other malignant epithelial neoplasms & melanomas	776	96.8 (94.2–98.2)	93.3 (90.1–95.5)	86.5 (81.4–90.3)
XID. Malignant melanomas	493	99.0 (96.1–99.8)	97.5 (94.3–99.0)	91.1 (84.7–95.0)
XII. Other & unspecified	24	77.4 (45.1–92.2)	72.2 (41.7–88.7)	58.3 (29.4–79.0)

a Includes all children who were diagnosed between January 1st 1983 and December 31st 2006, and were ‘at risk’ at some point between 1997 and 2006 (inclusive).

b *N* represents the total number at risk between 1997 and 2006 (inclusive).

c Includes cancers of benign or uncertain behaviour.

**Table 2:** Relative survival for Australian children diagnosed with cancer by diagnostic group and stage, 1997–2006^a^

		**Relative survival (95% confidence intervals)**		
**Diagnostic group and stage**	***N* ^b^**	**1 year**	**5 year**	**20 year**
II. Lymphomas				
Stage I	259	98.4 (93.8–99.6)	96.3 (91.3–98.5)	96.5 (91.2–99.1)
Stage II	259	99.2 (94.6–99.9)	96.9 (91.9–98.9)	94.5 (88.4–97.7)
Stage III	321	90.6 (85.1–94.1)	84.3 (77.9–89.0)	80.1 (71.6–86.3)
Stage IV	138	92.8 (83.5–96.9)	86.1 (74.8–92.6)	85.0 (73.1–92.1)
Unknown	243	87.1 (79.7–91.9)	85.2 (77.4–90.5)	85.0 (77.0–90.6)
IV. Neuroblastoma				
Stage I	104	98.7 (89.7–100.0)	95.6 (86.3–98.8)	96.0 (86.6–99.2)
Stage II	88	100.0 NA	90.9 (77.1–96.7)	91.3 (77.4–97.1)
Stage III	102	87.7 (74.4–94.4)	74.4 (59.1–84.7)	74.7 (59.3–85.1)
Stage IV	297	81.7 (75.5–86.5)	49.3 (42.0–56.2)	41.8 (33.8–49.7)
Unknown	59	91.1 (68.6–97.8)	91.2 (68.7–97.9)	86.9 (63.3–96.2)
VI. Renal tumours				
Stage I	190	98.7 (90.3–99.9)	94.8 (86.4–98.1)	90.6 (80.3–95.8)
Stage II	135	100.0 NA	93.2 (84.1–97.2)	93.6 (84.5–97.7)
Stage III	165	97.4 (89.8–99.4)	92.3 (83.5–96.5)	91.3 (82.0–96.1)
Stage IV	122	91.2 (81.3–96.0)	75.1 (62.9–83.7)	73.7 (61.3–82.8)
Unknown	71	84.8 (69.2–92.9)	84.9 (69.2–93.0)	85.4 (69.6–93.6)
IXA. Rhabdomyosarcomas				
Stages I/II	115	97.0 (88.2–99.3)	86.4 (75.3–92.7)	81.3 (66.5–90.2)
Stages III/IV	123	89.2 (77.6–95.0)	55.9 (42.5–67.4)	47.1 (34.8–58.6)
Unknown	105	91.2 (78.2–96.6)	68.9 (46.1–83.6)	66.3 (44.5–81.3)

Abbreviation: NA=not applicable.

a Includes all children who were diagnosed between January 1st 1983 and December 31st 2006, and were ‘at risk’ at some point between 1997 and 2006 (inclusive).

b *N* represents the total number ‘at risk’ between 1997 and 2006 (inclusive).

**Table 3:** Adjusted 5-year hazard ratios for children diagnosed with cancer in Australia by diagnostic group, sex, age at diagnosis, year of diagnosis and stage (where relevant), 1997–2006a,b,c

	**Sex**		**Age at diagnosis^d^**				**Year of diagnosis**		**Stage^e^**			
**Cancer type**	**Boys**	**Girls**	**<1 years**	**1–4 yrs**	**5–9 yrs**	**10–14 yrs**	**1992–1998**	**1999–2006**	**I**	**II**	**III**	**IV**
All cancers combined	1.00	0.91 (0.82–1.02)	1.39 (1.15–1.67)	1.00^*^	1.08 (0.93–1.24)	0.96 (0.84–1.11)	1.00^*^	0.84 (0.74–0.94)	—	—	—	—
I. Leukaemias	1.00^*^	0.76 (0.62–0.93)	4.75 (3.45–6.54)	1.00^**^	1.32 (1.02–1.71)	1.99 (1.53–2.58)	1.00^**^	0.59 (0.48–0.73)	—	—	—	—
IA Lymphoid leukaemias	1.00^*^	0.65 (0.50–0.85)	6.47 (3.93–10.63)	1.00^**^	1.42 (1.04–1.96)	2.11 (1.50–2.95)	1.00^**^	0.61 (0.47–0.80)	—	—	—	—
IB. Myeloid leukaemias	1.00	1.04 (0.72–1.49)	1.83 (1.06–3.15)	1.00	1.00 (0.61–1.63)	1.27 (0.79–2.05)	1.00^**^	0.52 (0.35–0.76)	—	—	—	—
II. Lymphomas	1.00	1.12 (0.65–1.94)	0.92 (0.11–7.78)	1.00	1.09 (0.54–2.21)	0.78 (0.39–1.54)	1.00	0.61 (0.36–1.04)	1.00^*^	0.83 (0.21–3.21)	4.41 (1.65–11.78)	3.74 (1.22–11.47)
III. Tumours of the CNS^f^	1.00	1.01 (0.83–1.23)	2.12 (1.53–2.93)	1.00^**^	0.83 (0.65–1.06)	0.62 (0.47–0.81)	1.00	0.98 (0.79–1.21)	—	—	—	—
IV. Neuroblastoma	1.00	1.23 (0.84–1.79)	0.26 (0.15–0.45)	1.00^**^	1.47 (0.88–2.48)	0.63 (0.19–2.04)	1.00	0.84 (0.57–1.25)	1.00^**^	2.18 (0.48–9.96)	6.08 (1.68–22.00)	12.73 (3.95–40.96)
VI. Renal tumours	1.00	0.77 (0.39–1.52)	4.69 (2.08–10.55)	1.00^*^	1.55 (0.65–3.69)	2.08 (0.53–8.23)	1.00	0.91 (0.45–1.83)	1.00^*^	1.28 (0.33–4.89)	2.09 (0.57–7.72)	6.01 (1.97–18.37)
VII. Hepatic tumours	1.00	0.60 (0.23–1.57)	0.60 (0.17–2.16)	1.00^*^	3.03 (0.96–9.57)	3.01 (1.03–8.79)	1.00	1.12 (0.44–2.87)	—	—	—	—
VIII. Malignant bone tumours	1.00	0.78 (0.50–1.22)		1.00^*^	1.85 (1.09–3.13)		1.00	0.81 (0.51–1.28)	—	—	—	—
IX. Soft-tissue sarcomas	1.00	0.81 (0.53–1.23)	1.27 (0.53–3.04)	1.00	1.11 (0.62–2.00)	1.62 (0.96–2.73)	1.00	1.01 (0.65–1.57)	—	—	—	—
IXA. Rhabdomyosarcomas	1.00	0.83 (0.45–1.54)		1.00^*^	1.07 (0.51–2.26)	3.12 (1.49–6.54)	1.00	1.21 (0.62–2.37)	1.00^**^	4.69 (2.11–10.43)		
X. Germ-cell tumours^f^	1.00	1.52 (0.66–3.50)	2.17 (0.62–7.57)	1.00	3.20 (0.87–11.78)	1.39 (0.40–4.85)	1.00	0.78 (0.34–1.82)	—	—	—	—
XI. Other malignant epithelial neoplasms & melanomas	1.00	0.61 (0.27–1.40)		1.00	1.90 (0.39–9.31)	0.67 (0.15–3.06)	1.00	0.99 (0.42–2.34)	—	—	—	—

a Includes survival upto 5 years after diagnosis, so the ‘at risk’ period of 1997–2006 means that children who were diagnosed between January 1st 1992 and December 31st 2006 have the potential to be included in the analysis.

b Model for retinoblastoma did not converge because of insufficient deaths and so is not included in this table.

c ^**^: highly significant group effect (*P*<0.001); ^*^: significant group effect (0.001<*P*<0.05).

d Insufficient numbers of cases aged <1 meant that children <1 years and 1–4 years were combined for rhabdomyosarcomas and other malignant epithelial neoplasms and melanomas, and 0–9 years combined for malignant bone tumours.

e Cases with unknown stage were included in the models, but separate results are not presented in the tables.

f Includes cancers of benign or uncertain behaviour.
